# Research Hot Spots and Trends on Melatonin From 2000 to 2019

**DOI:** 10.3389/fendo.2021.753923

**Published:** 2021-11-30

**Authors:** Yan Meng, Zhengbo Tao, Siming Zhou, Wacili Da, Lin Tao

**Affiliations:** Department of Orthopaedics, First Hospital of China Medical University, Shenyang, China

**Keywords:** melatonin, bibliometrics, pineal gland, hotspots, clinical applications

## Abstract

Research on melatonin remains one of the major hot spots in the field of disease treatment, but relevant data are numerous. The purpose of this study was to quantitatively and qualitatively analyze the progress of melatonin research through the method of bibliometrics and to predict hot spots and trends in melatonin research. This study retrieved all the studies on melatonin from 2000 to 2019 in the Web of Science and PubMed and analysed the publishing trends in the literature on a bibliometric online analysis platform and CiteSpace software. The research results were also visually analysed to summarize melatonin research hot spots through gCLUTO and pubMR. The study retrieved a total of 20,351 publications, of which the number of US publications ranked first, accounting for 21.46%, with the greatest impact (centrality = 0.31). The University of Texas Health Science Center at San Antonio and Harvard University had the highest average number of citations at 43.19 and 33.96, respectively. *Journal of Pineal Research* had the highest average number of citations in 2,993 journals. Professor Reiter made the largest contribution to this area. We further analysed 100 highly cited articles for clinical applications and ongoing related clinical drug trials based on the first hot spot. We systematically analysed melatonin for nearly 20 years while predicting the main research trends in the future, which may provide new directions and ideas for melatonin research. The structure and normal physiological functions of melatonin have been intensively studied in the past few years. And clinical application research and target of melatonin treatment for different diseases and target-based drug design will certainly become the focus of melatonin research.

## Introduction

Melatonin, first discovered in the late 1950s, is a pleiotropic neurohormone secreted by the pineal gland (1). The production of melatonin is regulated by the circadian rhythm with light/dark signals, and light will inhibit the production of melatonin; therefore, the concentration in the daytime is low, whereas the concentration at night will reach a peak (2–4). With the continuous progress in melatonin research, the structure and function of melatonin and its receptors have also been gradually revealed, and an increasing number of scientific researchers have devoted themselves to this research field with fruitful results (5, 6). Melatonin can regulate many physiological processes, such as sleep rhythms, blood pressure, immune function, temperature rhythms, physiological activity rhythms, reproductive function, cell differentiation and proliferation, bone metabolism and fat metabolism, memory formation, and oxidative stress, and it is involved in the inhibition of cancer and inflammatory processes (7–18). Thus, the clinical application value of melatonin has received increasing attention.

Bibliometric analysis has become the best tool for assessing the status of and trends in a particular research field. Compared with systematic review or meta-analysis which usually pays more attention to integration and secondary analysis of the content of each included study using statistical methods, bibliometric analysis can include much more studies, analyze the literature without focusing on the content in detail. And there is specialized software to analyze the including papers comprehensively along with the visual presentation. This analysis method objectively summarizes the number of publications produced by various countries, institutions, journals and authors and their contributions to a certain scientific field *via* qualitative and quantitative analysis, and it forecasts the research trends or hot spots. The cooperation trends are conducive to discovering which researchers or institutions are leading and authoritative in the research field, which can provide more valuable references for later researchers. Based on this, they can get new inspirations and further study the field in more depth. In addition, bibliometrics is also applied to help guide the development of clinical policies and guidelines on many diseases, such as sepsis, sacral fracture, and childhood leukemia (19–21). However, there have been many studies on melatonin in recent years, with no bibliometric analysis on melatonin research, and even less attention has been focused on the prediction of research hot spots. Our previous research has predicted the research trends in post-menopausal osteoporosis through the biclustering method and demonstrated that biclustering not only can process the global information but also can allow the rows and columns of the matrix to be processed simultaneously so that it can detect local messages more efficiently when encountering high-dimensional data; furthermore, biclustering has great advantages in exploring key areas of research and the related representative literature (22, 23). Based on the previous foundation, our team developed a new original tool, pubMR, which is an R package designed for text mining of PubMed abstracts to perform hot spot analyses (24). Additionally, pubMR provides some highly customized metrics to evaluate and visualize results for downstream analysis, which greatly improves the efficiency and credibility of the research.

Considering the vast plethora of studies published on melatonin, there is a dire need to investigate the status of and trends in this topic. There have been few bibliometric studies on melatonin, and those few paid more attention to studying published information than future research trends (25–28). This study aimed to conduct a comprehensive investigation of the current academic literature and clinical applications of melatonin and to predict the developmental trend in melatonin research over the next decades through citation analysis combined with dual clustering analysis.

## Materials and Methods

### Data Extraction and Collection

We used the TI = (melatonin) AND Language = English search strategy on the Web of Science to collect all the original articles and reviews on melatonin research published from 2000 to 2019.

The Medical Subject Headings (MeSH) term is a type of standard vocabulary from the National Center for Biotechnology Information (NCBI) of the National Library of Medicine (NLM) that can be adapted to perform continuous co-word cluster analysis and reflect the main topic of the literature (29). We also conducted a similar online search in PubMed based on the screening criteria of “melatonin”[Mesh]. All the downloading processes were completed within one day, on April 2, 2020, to avoid errors caused by frequent database updates. Completed phase II clinical trials were obtained from ClinicalTrials.gov (https://clinicaltrials.gov/).

All data were independently collected by two authors (ZBT and SMZ) with an agreement rate that eventually reached 0.93 (30). The data obtained from the Web of Science were converted into text format and imported into CiteSpace V5.5.R1 SE, 64-bit, for analysis. Then, we used the Online Analysis Platform of Literature Metrology (http://bibliometric.com/) to perform a bibliometric analysis. The data obtained from PubMed were uploaded to pubMR for progressive analysis.

### Bibliometric Analysis

We analysed the data; summarized the number of articles published by different countries, institutions, journals, and authors; and obtained the impact factor and H index of all publications. Moreover, the annual numbers of and growth trend in publications from various countries or regions were analysed through online platforms. The collaborations among countries, institutions and authors were identified through CiteSpace. A co-occurrence analysis of the keywords was performed to predict research fronts and new trends. The method of “time slicing” was also performed in CiteSpace, as described in our previous publication (22). Depending on the purpose of our analysis, we chose different nodes, for which the size represents the citation count or the quantity of publications.

### Co-Word Biclustering Analysis

We carried out biclustering analysis of major MeSH terms/MeSH subheadings and selected publications to explore the hot spots in melatonin research. A binary matrix was constructed through pubMR and gCLUTO version 1.0 (Graphical Clustering Toolkit). The major MeSH terms/MeSH subheadings were set as rows, with the source articles as columns. The details are accessible in our published research (22). We conducted mountain and matrix visualization on the results of biclustering, which can reflect relationships between major MeSH terms/MeSH subheadings and the source articles.

### Citation Analysis

The Web of Science Core Collection database was retrieved to obtain articles related to the clinical treatment of melatonin-related diseases published from 2000 to 2019 on June 16th after identifying the hot spots, with melatonin and clinical treatment as the retrieval subjects, and the language was limited to English. The search results were sorted according to the citation frequency from high to low, and the top 100 articles were selected for analysis. The analysis content includes citation frequency, author, institution, country, research direction, article type, publication journal, and publication year. Next, we read the full text of these 100 articles, summarizing the focus of melatonin clinical treatment and exploring potential new therapeutic applications. To better grasp the clinical progress of melatonin, we also collated all the completed phase II clinical trials of melatonin.

## Results

### Publishing Trend

**Figure 1:**
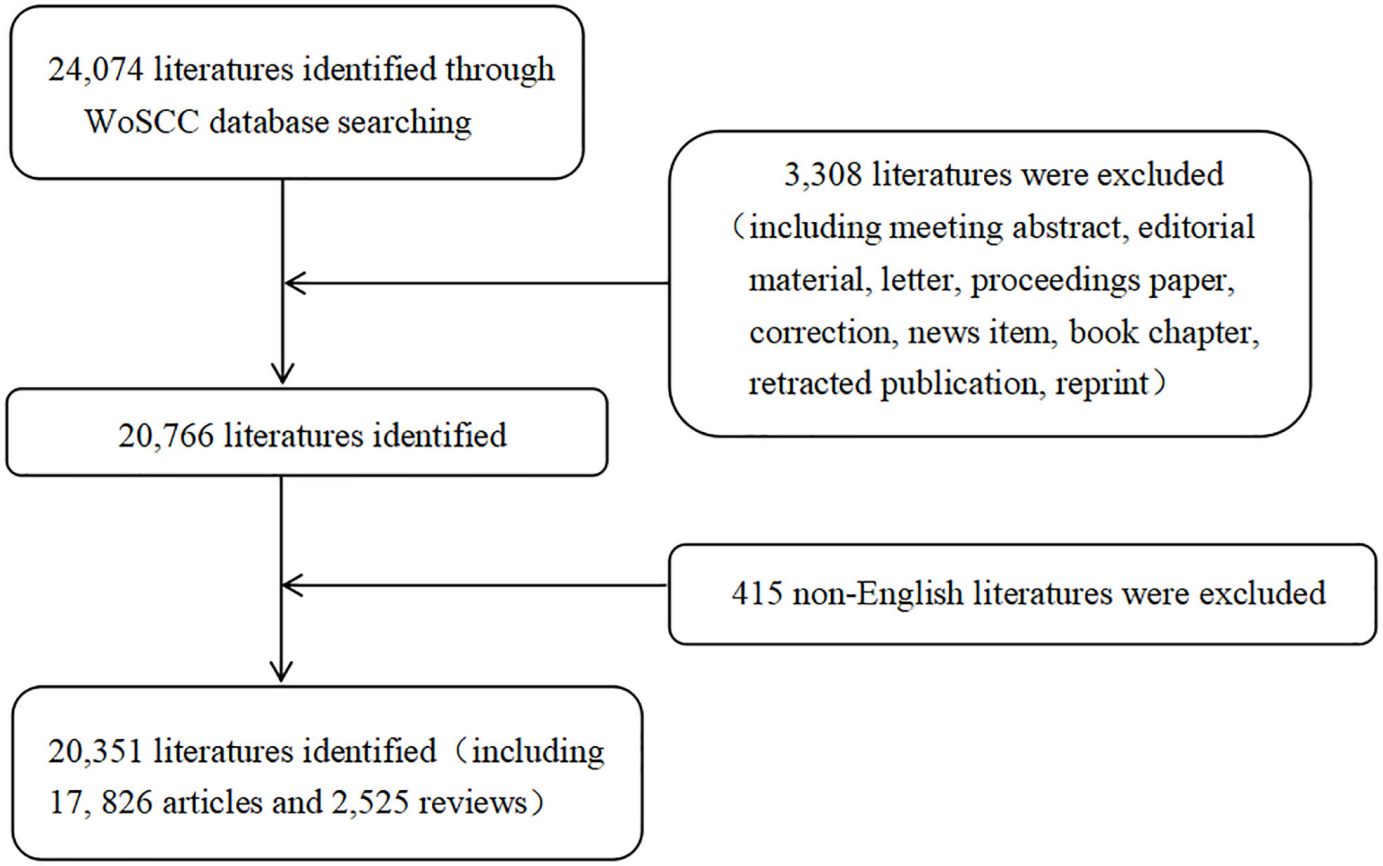
Flow chart of literature collecting.

**Figure 2:**
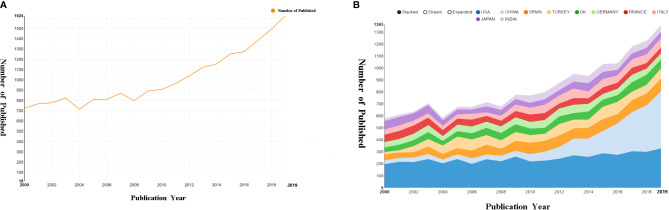
Publication trend. **(A)** Number of publications per year. **(B)** Number of publications in the top 10 countries.

As shown in **Figure 1**, a total of 20,351 publications (17,826 articles and 2,525 reviews) were collected that met our inclusion criteria. Publications related to melatonin research showed an increasing trend from 2000 to 2019 (an overall increase from 726 in 2,000 to 1,624 in 2019, **Figure 2A**).

### Contributions of Countries and Institutions

**Figure 3:**
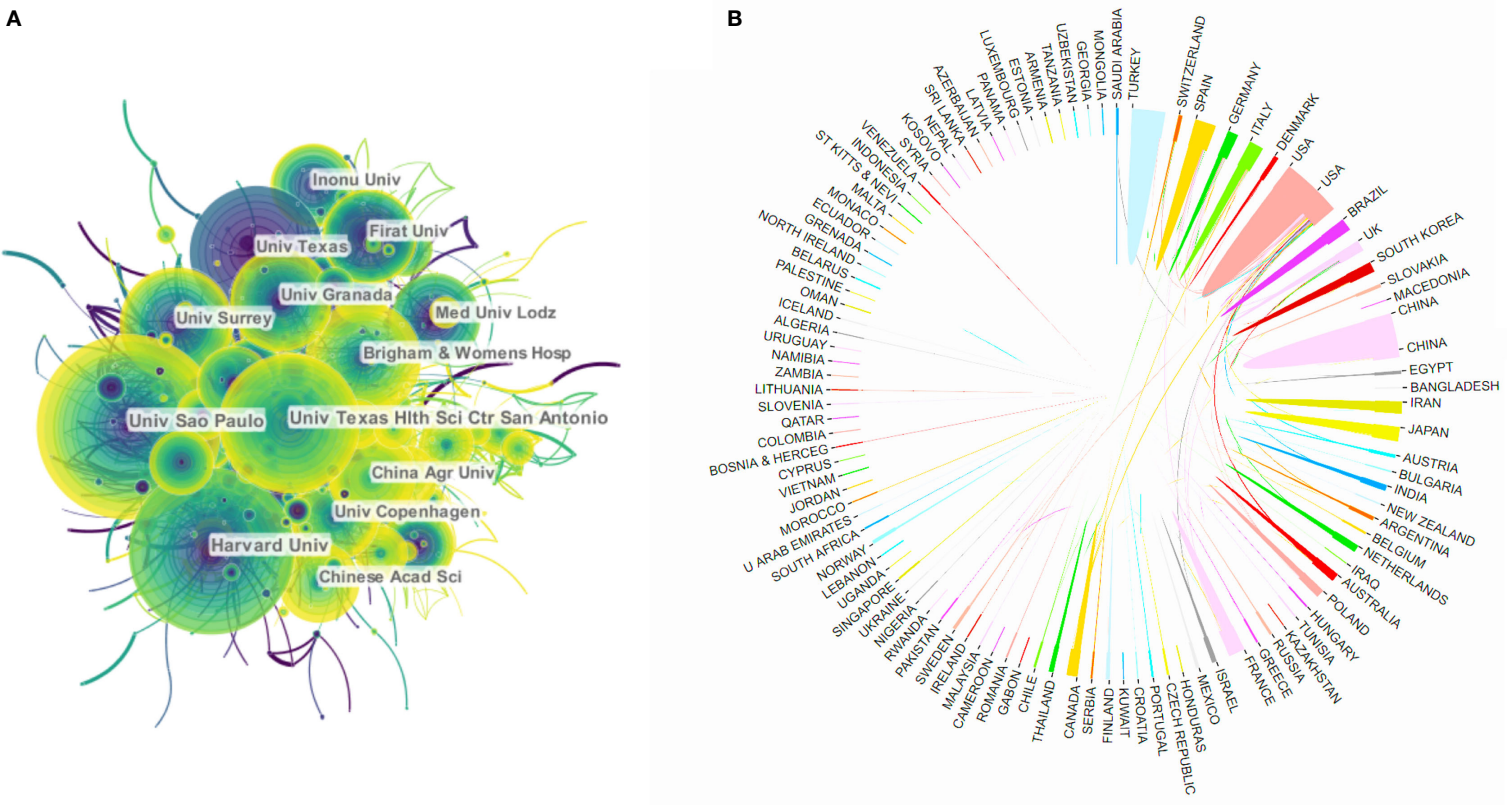
Cooperation among different countries and institutions. **(A)** Density map of cooperation among institutions. **(B)** Cooperation among different countries.

From 2000 to 2019, at least 62 countries and regions published articles about melatonin (**Figure 2B**). The United States (4,978) published the most among the countries, followed by China (2,556), Spain (1,535), Turkey (1,424), and the United Kingdom (1,164). Centrality is a vital indicator reflecting the importance of a network node. A node with high centrality reflects that the node has great influence. We found that the influence of the United States is highly prominent, with centrality = 0.31, followed by France, with centrality = 0.22, which ranks 7th in the number of publications (**Table 1**). American universities among the top 10 institutions have posted a significant number of papers. In addition, the University of Texas Health Science Center at San Antonio and Harvard University have the highest average number of citations at 43.19 and 33.96, respectively (**Table 1**). The density map (**Figure 3A**) reflects the cooperative relationship between institutions, with density = 0.0117, which shows that the research of various institutions is relatively scattered, and the degree of cooperation is relatively low and needs to be strengthened. **Figure 3B** reveals that there is an absence of academic exchanges between countries with abundant publications and countries with weak publications.

**Table 1:** The top 10 countries/regions and institutions contributing to publications in melatonin research.

Rank	Country/Region	Article Counts	Centrality	Institutions	Article Counts	Total number of citations	Average number of citations	Total number of first author	Total number of first author citations	Average number of first author citations
1	USA	4978	0.31	Univ Sao Paulo	445	6073	13.65	193	3206	16.61
2	China	2556	0.08	Harvard Univ	435	14774	33.96	85	2993	35.21
3	Spain	1535	0.11	Inonu Univ	379	2755	7.27	92	752	8.17
4	Turkey	1424	0.02	Univ Texas Hlth Sci Ctr San Antonio	340	14684	43.19	56	4442	79.32
5	UK	1164	0.05	Univ Granada	321	9308	29	123	4150	33.74
6	Germany	1101	0.16	Firat Univ	309	3095	10.02	107	1411	13.19
7	France	1094	0.22	Med Univ Lodz	296	4638	15.67	124	1619	13.06
8	Italy	1084	0.11	Brigham & Women’s Hosp	271	4730	17.45	106	2798	26.4
9	Japan	1077	0.05	Kaohsiung Chang Gung Mem Hosp	255	2375	9.31	47	440	9.36
10	India	886	0.04	Suleyman Demirel Univ	254	1868	7.35	83	654	7.88

### Journals Publishing Melatonin Research

The 20,351 publications covered 2,993 journals, the top 10 most popular journals published a total of 3,732 publications (18.34%), and the number of articles published in *Journal of Pineal Research* far exceeded that of other journals, accounting for more than 8.38% (1,705/20,351) (**Table 2**). Three journals had an IF> 3: *Journal of Pineal Research* (15.221), *International Journal of Molecular Sciences* (4.183), and *Life Sciences* (3.448). The above three journals belong to Q1, which is based on the JCR 2018 standard.

**Table 2:** The top 10 most active journals that published articles in melatonin research.

Rank	Journal title	Percentage (N/20,351)	IF (2018)	Quartile in category (2018)	H-index	Article Counts	Total number of citations	Average number of citations
1	JOURNAL OF PINEAL RESEARCH	8.43%	15.221	Q1	113	1715	60052	35.02
2	CHRONOBIOLOGY INTERNATIONAL	2.15%	2.562	Q2	87	438	4603	10.51
3	PLOS ONE	1.24%	2.776	Q1	268	252	2142	8.5
4	NEUROENDOCRINOLOGY LETTERS	1.16%	0.698	Q3	58	236	2640	11.19
5	JOURNAL OF BIOLOGICAL RHYTHMS	1.07%	2.473	Q1	91	217	4106	18.92
6	INTERNATIONAL JOURNAL OF MOLECULAR SCIENCES	1.02%	4.183	Q1	114	207	2064	9.97
7	BIOLOGICAL RHYTHM RESEARCH	0.88%	0.773	Q3	30	179	335	1.87
8	LIFE SCIENCES	0.81%	3.448	Q1	150	165	2834	17.18
9	NEUROSCIENCE LETTERS	0.80%	2.173	Q3	155	162	2089	12.9
10	GENERAL AND COMPARATIVE ENDOCRINOLOGY	0.79%	2.445	Q1	105	161	1561	9.7

*Journal of Pineal Research* published 1,715 articles in the field of melatonin, ranking first. In the past 20 years, the total number of citations of these articles has reached 60,052, with an average of more than 35 citations per article. The number of publications of other journals, including *Chronobiology International*, *PloS One* and *Neuroendocrinology Letters*, was ranked in the back of *Journal of Pineal Research* in order. Moreover, *Journal of Pineal Research* also has the highest impact factor among these journals. Thus, these findings imply that *Journal of Pineal Research* has extraordinary authority in melatonin research and that the journal mentioned above may publish more articles that can advance the field in the future. In addition, authors who have made outstanding contributions in the field of melatonin have also published a large number of high-quality articles in these journals. These journals have published a large number of melatonin-related articles, have a wide influence and have become an important reference for melatonin researchers. There is no doubt that these journals have laid a solid foundation for future in-depth research on melatonin.

### Contributions of Authors to Melatonin Research

A total of 52,615 authors were filtered out in this study, and the top 10 authors who contributed the most to melatonin research are listed in **Table 3**. Two scholars, Dunxian Tan from UT Health San Antonio and Rüdiger Hardeland from the University of Göttingen, had the highest average number of citations of 92.5 and 69.54, respectively, which demonstrated that they have made great achievements in the study of melatonin and that their publications are of great academic value. Russel J Reiter (638) was the most productive author, followed by Dunxian Tan (218), Darío Acuña-Castroviejo (124) and Philippe Delagrange (121) (**Table 3**). These researchers have become influential experts in melatonin research, have reported a substantial amount of research, and lead the development of melatonin research in recent years.

**Table 3:** The top 10 most productive authors in melatonin research.

Rank	Author	Article Counts	Total number of citations	Average number of citations	First author counts	First author citations counts	Average first author citations counts	Corresponding author counts	Corresponding author citation counts
1	Reiter, RJ	638	34666	54.34	60	6968	116.13	141	15097
2	Tan, DX	218	20164	92.5	26	3543	136.27	10	912
3	Acuna-Castroviejo, D	124	5414	43.66	8	780	97.5	60	2974
4	Delagrange, P	121	3118	25.77	2	71	35.5	3	216
5	Cardinali, DP	108	4195	38.84	20	677	33.85	65	2012
6	Escames, G	98	4277	43.64	10	486	48.6	12	183
7	Pevet, P	98	2115	21.58	8	324	40.5	20	422
8	Haldar, C	94	751	7.99	13	132	10.15	78	633
9	Hardeland, R	87	6050	69.54	31	2697	87	44	3250
10	Zhang, Y	86	852	9.91	19	107	5.63	6	24

### Hot Spot Analysis

**Figure 4:**
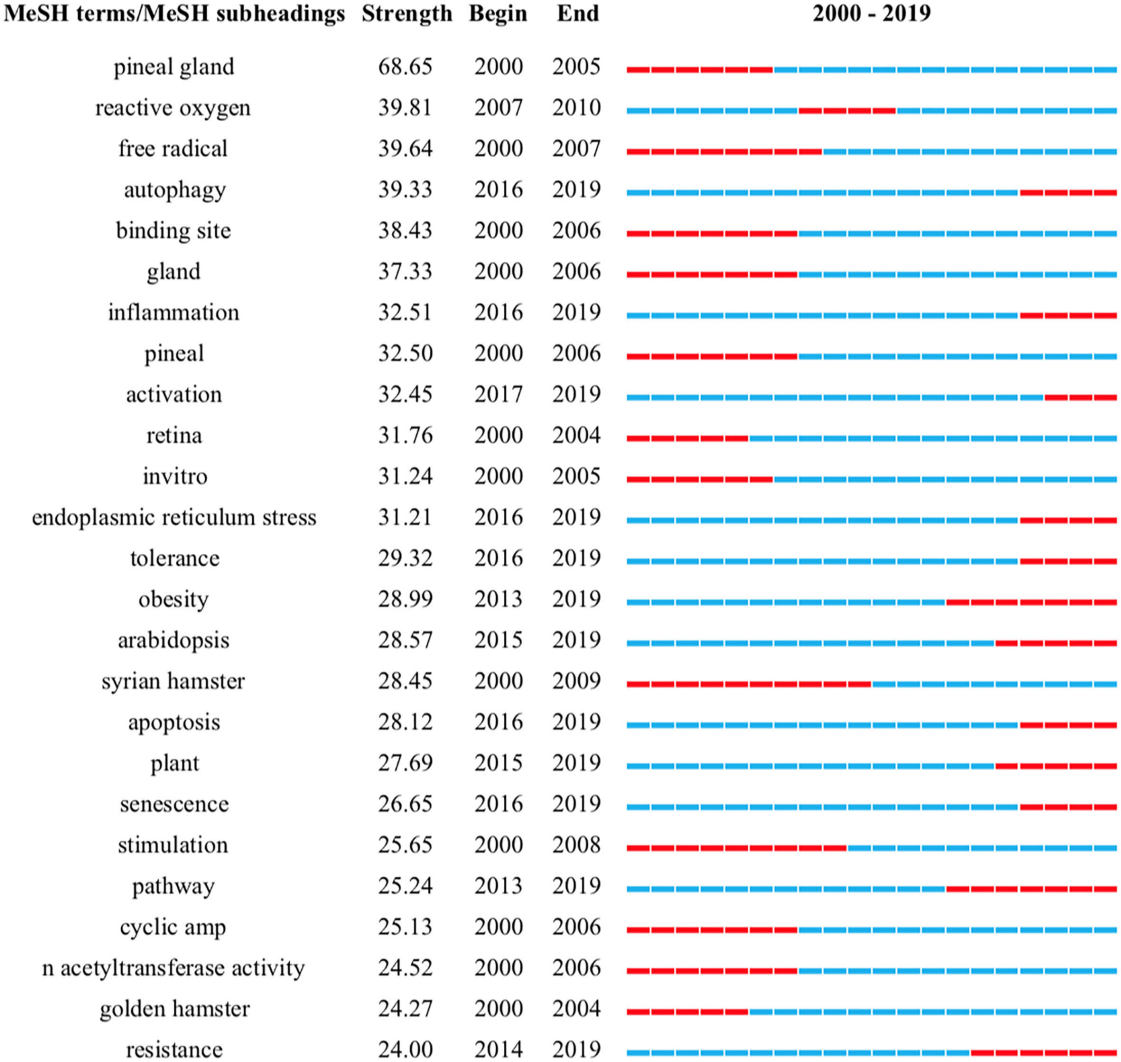
The top 25 Mesh terms and their outbreak time (Strength reflects the frequency of occurrence of the keyword).

**Figure 5:**
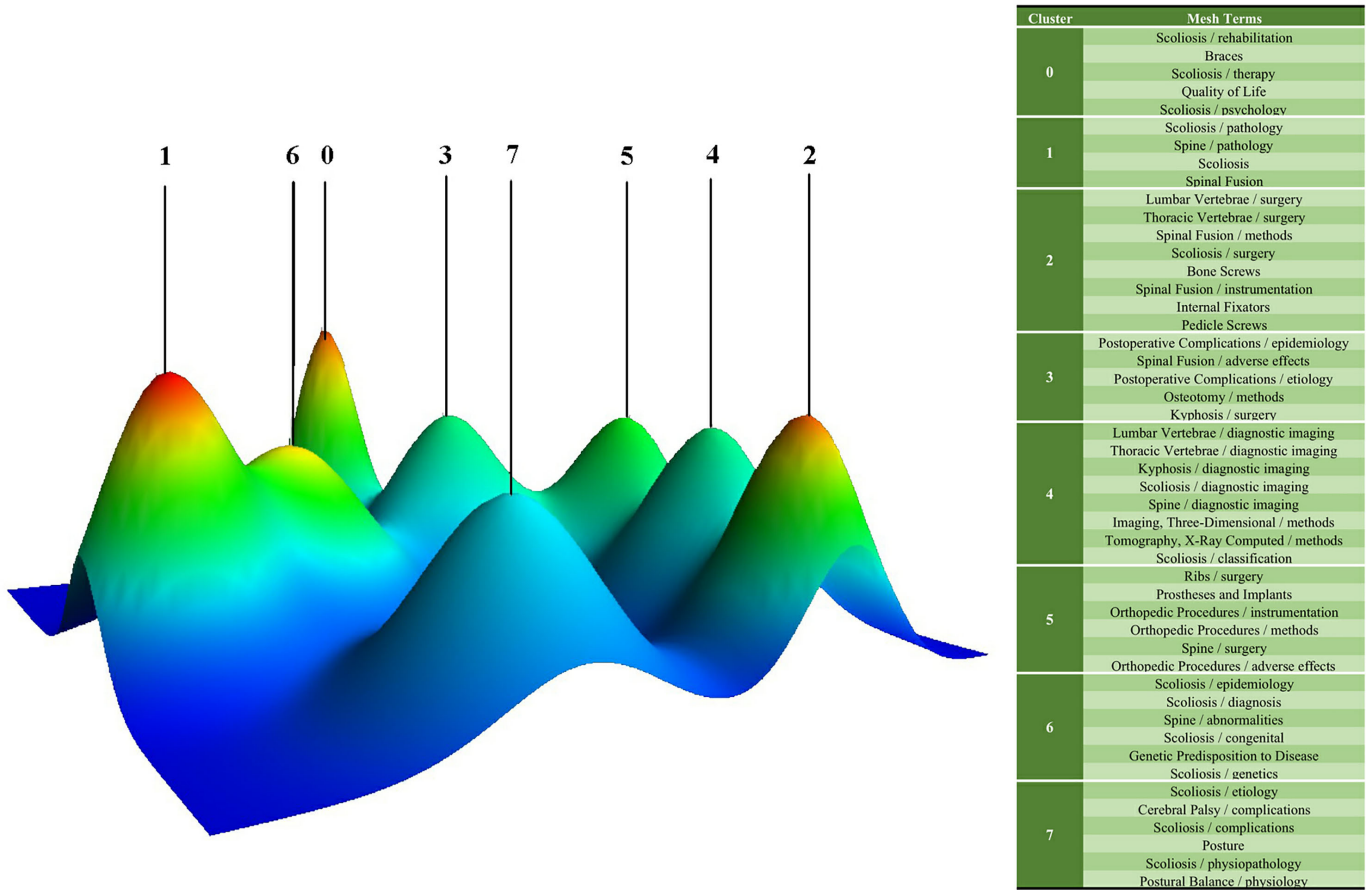
Mountain visualization of the eight hot spots by biclustering.

**Figure 6:**
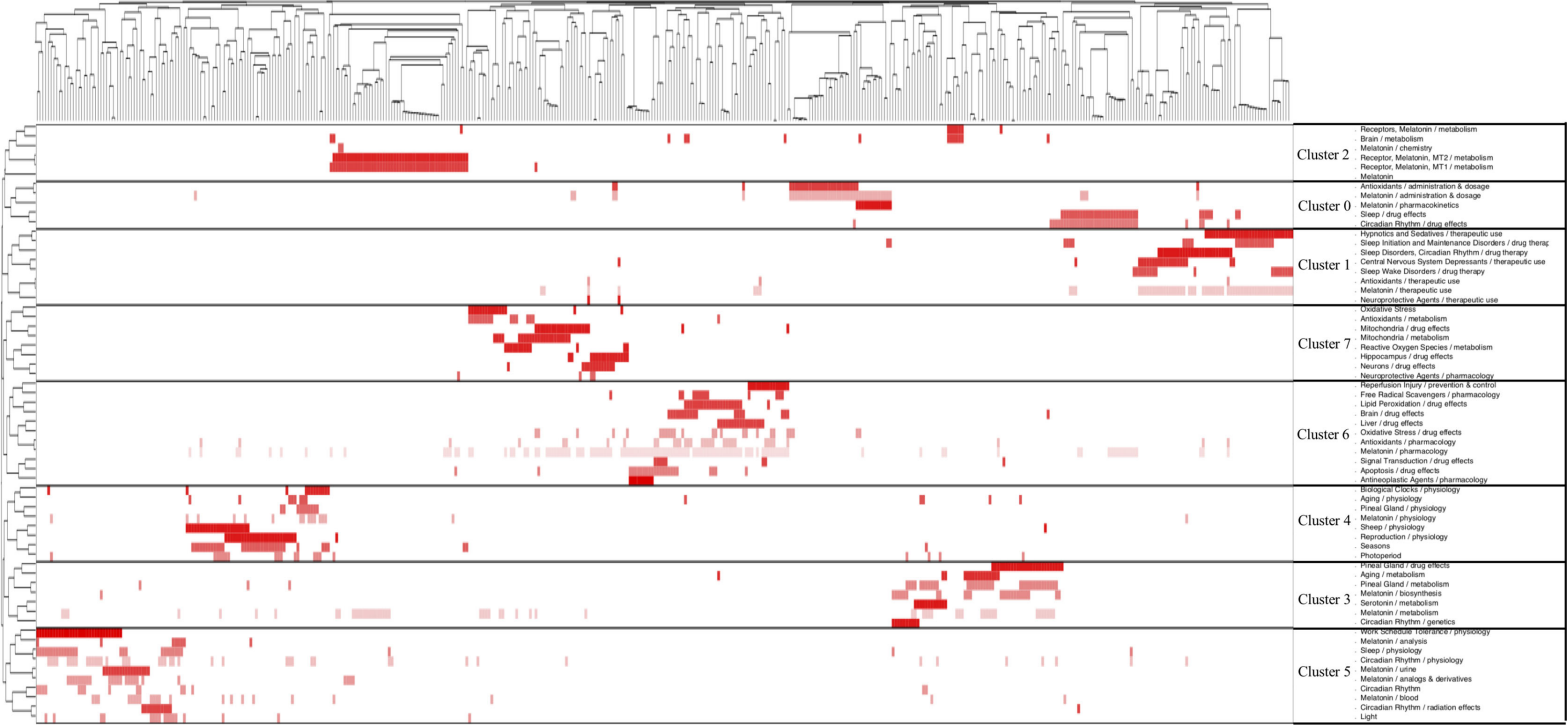
Visualized matrix of the MeSH terms and source articles.

A total of 12,563 major MeSH terms/MeSH subheadings were collected with a cumulative frequency of 56,099. After considering the H index, terms with a frequency of more than 66 were defined as extreme frequency terms, and sixty-three terms extracted from publications accounting for 34.41% (19,303/56,099) are shown in **Table S1**. **Figure 4** reflects the concentration time period of the top 25 terms and the change in hot spots with time from 2000 to 2019. The eight clusters (0-7) identified by biclustering were visualized in mountain form to indicate the quantity of extremely frequent terms and in matrix form to present the association between the terms and the source articles (**Figures 5**, **6**). The rows of the initial matrix were reset through gCLUTO to facilitate similar row convergence in a single cluster. Each cluster was divided with a black horizontal line (**Table 4**).

**Table 4:** Highly frequent major MeSH terms/MeSH subheadings -source articles matrix (localized).

No.	Major MeSH terms/MeSH subheadings	Pubmed Unique Identifiers of source articles			
		10561083	10600680	10607943	31914295
1	Melatonin/pharmacology	1	0	1	0
2	Melatonin/metabolism	0	0	0	0
3	Melatonin/therapeutic use	0	0	0	0
4	Circadian Rhythm/physiology	0	0	0	0
62	Melatonin	0	0	0	1
63	Work Schedule Tolerance/physiology	0	0	0	0

### The Top 100 Most-Cited Articles on Clinical Treatment of Melatonin-Related Diseases

According to the first hot spot clinical application, the 100 most-cited articles on the application of melatonin in the treatment of clinical diseases were further analysed. The results showed that as of June 16, the number of citations of these 100 articles ranged from 98 to 467, and the cumulative number of citations was 16,370 (**Table S2**). The year with the most highly cited articles published was 2007, with 12 records; the author who contributed the most was Reiter RJ, with 13 articles; the institution that produced the most was UNIV TEXAS (9); the most influential country was the USA (49, nearly half of the total); most of the types of articles were articles (52) and reviews (47); *JOURNAL OF PINEAL RESEARCH* contributed 14 of these articles, more than double the number two journal, *SLEEP* (6), with absolute authority; and the most researched direction was NEUROSCIENCES NEUROLOGY (53), followed by ENDOCRINOLOGY METABOLISM and PHYSIOLOGY, both with 18 records (**Table 5**). After analyzing the context of the 100 most-cited articles, we found that melatonin is currently mainly used in the treatment of neurological and psychiatric diseases and antitumour therapy. There were 30 studies that completed phase II clinical trials that also focused on central nervous system diseases and antitumour treatment (**Table S3**).

**Table 5:** Publication of the 100 most-cited articles on melatonin research.

Classes	Items	Number	Classes	Items	Number
Publication Year	2017	3	Institution	UNIV MESSINA	4
	2016	1		UNIV TORONTO	4
	2015	3	Country/Region	USA	49
	2014	3		FRANCE	16
	2013	6		CANADA	13
	2012	3		ENGLAND	13
	2011	6		ITALY	11
	2010	9		GERMANY	9
	2009	8		NETHERLANDS	9
	2008	6		SPAIN	8
	2007	12		ISRAEL	6
	2006	5		PEOPLES R CHINA	6
	2005	8	literature types	ARTICLE	52
	2004	9		REVIEW	47
	2003	7		PROCEEDINGS PAPER	6
	2002	4		EDITORIAL MATERIAL	1
	2001	4	Journals	JOURNAL OF PINEAL RESEARCH	14
	2000	3		SLEEP	6
Author	REITER RJ	13		JOURNAL OF CLINICAL PSYCHIATRY	5
	TAN DX	7		BRAIN	3
	BARBERI I	4		JOURNAL OF SLEEP RESEARCH	3
	CARDINALI DP	4		NEUROLOGY	3
	GITTO E	4		ARCHIVES OF GENERAL PSYCHIATRY	2
	LAUDON M	4		INTERNATIONAL JOURNAL OF MOLECULAR SCIENCES	2
	ZISAPEL N	4		JOURNAL OF BIOLOGICAL RHYTHMS	2
	KASPER S	3		PEDIATRICS	2
	TRIMARCHI G	3	Research Direction	NEUROSCIENCES NEUROLOGY	53
	ARNULF I	2		ENDOCRINOLOGY METABOLISM	18
Institution	UNIV TEXAS	9		PHYSIOLOGY	18
	UNIV CALIF SAN DIEGO	6		PSYCHIATRY	14
	MAYO CLIN	5		PHARMACOLOGY PHARMACY	13
	UNIV CALIF LOS ANGELES	5		PSYCHOLOGY	7
	COLUMBIA UNIV	4		ONCOLOGY	6
	PONTIFICIA UNIV CATOLICA ARGENTINA	4		PEDIATRICS	6
	TEL AVIV UNIV	4		BIOCHEMISTRY MOLECULAR BIOLOGY	5
	UNIV ILLINOIS	4		GENERAL INTERNAL MEDICINE	4

## Discussion

Melatonin, as an endogenous hormone in the human body, has a strong relationship with the occurrence, development and treatment of diseases. Our statistical and quantitative analysis found a significant improvement in research on melatonin from 2000 to 2019, and an increasing number of researchers are entering this research field. The United States has an absolute leading position in the field of melatonin research. Many institutions and authors who have contributed the most in this field also come from the United States. The United States has advanced equipment, a high-quality experimental environment, good clinical trial conditions, and more professional talent, far ahead of other countries. However, there is less cooperation among various countries, and research results cannot be shared efficiently, which seriously affects the world’s overall understanding of melatonin and research efficiency. If cooperation between countries can be strengthened, considerable duplication or invalid work can be avoided, and research on melatonin will surely make more progress. Although studies on melatonin have been highly extensive, they are relatively chaotic and lack hot spot analysis. In this study, we found and interpreted 8 hot spots in melatonin research and analysed the 100 most-cited articles in the field of clinical treatment of melatonin-related diseases to predict future research trends.

Cluster 0 is related to the clinical application of melatonin. At present, only a few countries have officially applied melatonin to clinical practice, and most of these treatments are limited to those of sleep disturbances with melatonin a health product; however, with the continuous deepening of clinical studies on melatonin, melatonin is being tested by scholars for a variety of diseases, and a large number of clinical trials are going hand in hand, including those of sepsis, hypertension etc. (31–34). As a physiological natural hormone, melatonin is undoubtedly more suitable for the human body than chemically synthesized drugs. Indeed, melatonin is a drug candidate with great therapeutic potential that cannot be ignored. Therefore, further exploration of the potential clinical application of melatonin in different diseases, clarification of the specific targets of melatonin action, and development of new drugs based on the efficacy of melatonin for clinical disease treatment will be of great significance and are bound to become hot spots in future melatonin research.

Given the important role of melatonin clinical application in melatonin studies, we analysed 100 highly cited articles. The results showed that the most frequently cited field, which indicated the mainstream field most pursued by scholars, was the therapeutic application of melatonin in neuropsychiatric diseases and cancer, which was consistent with the focus of our collected clinical trials. Studies have shown that melatonin can effectively protect against age-related neurodegenerative diseases, such as Alzheimer’s disease (AD) and Parkinson’s disease (PD) (35, 36), and is expected to be used in neuroprotective treatment of cerebral trauma and ischemia-reperfusion after stroke (37); melatonin has been widely applied to treat sleep disturbances, especially sleep disorders caused by jet lag and melatonin reduction in the elderly, because melatonin can adjust circadian rhythms; and melatonin has a strong relationship with mental illnesses such as depression, which may be related to its adjustment of circadian rhythms to affect sleep (38). There are many ongoing clinical trials to confirm the effect of melatonin-related drugs on depression, such as agomelatin, which is a melatonin analogue, that have made some progress (39). Melatonin also plays a role in treating tumors. Melatonin can control tumor progression and can also be used as a protective agent for other tissues after chemotherapy and radiotherapy to reduce physical damage (40, 41). To our surprise, the results also indicated that melatonin has the potential to treat osteoporosis (42). Recent studies have found that osteoporosis is associated with circadian rhythm disorders, and melatonin can regulate circadian rhythm disorders, thereby correcting metabolic abnormalities. Melatonin also improves bone protection through antioxidative stress and anti-inflammatory effects, reducing bone loss. Although melatonin has the potential to treat osteoporosis, there are few highly cited studies on this topic, and there is still substantial room for research and clinical transformation. Therefore, we predict that the effects of melatonin on osteoporosis will grow into a new hot spot for the clinical application of melatonin in the future. Determining the direct-acting targets will be a breakthrough in clinical translation. Although melatonin is effective for many diseases, long-term ingestion of exogenous melatonin may inhibit the secretion of melatonin in the brain, disrupt circadian rhythms, and cause other problems. Therefore, the usage, dosage and safety of melatonin and synthetic melatonergic drugs need to be further explored through more clinical trials.

Cluster 1 is related to treatment for sleep disorders. The levels of melatonin secretion are closely related to sleep quality at night. As an important hormone that regulates circadian rhythms, disorders of melatonin secretion seriously affect sleep quality and cause sleep disturbance. Either endogenous or exogenous melatonin will break the irregular circadian rhythm, reduce the effect of promoting wakefulness, and restore the normal sleep rhythm. There is already evidence that melatonin has therapeutic effects on sleep disorders, such as insomnia, and diseases caused by circadian rhythm alterations. Melatonin also greatly helps to treat the delayed onset of sleep due to jet lag or shift work disorder. As the effect of melatonin on sleep disorders is clear, more future research should explore which types of sleep disorders melatonin is specifically suitable for. Moreover, systematic and comprehensive studies on the duration, dosage, dosage form, effective population, and long-term efficacy and safety of treatment should be further promoted (43–46).

Cluster 2 is related to the effect of the melatonin receptor on metabolism. The mechanism of action of melatonin is divided into receptor-dependent pathways and receptor-independent pathways. The melatonin receptors MT1 and MT2 are G protein-coupled receptors. The melatonin receptor has a high affinity for melatonin. Melatonin treatment of circadian-rhythm-disorder-related diseases, such as sleep disorders, bone metabolism, drug abuse and cancer, occurs through the mechanism of receptor-dependent pathways; the non-receptor-dependent pathway of melatonin is reflected in its role in protecting nerves. Recently, Linda C Johansson first analysed the crystal structure of MT1 and MT2 and revealed the molecular basis of melatonin action, which paved the way for melatonin pharmacological research. Different subtypes of melatonin receptors have different effects. However, melatonin has no subtype selectivity. Therefore, designing selective drugs for different subtypes will be a major entry point of melatonin to be made into drugs and used in clinical practice. Future research should focus on the analysis of groups that exert drug effects at the molecular level, the relationship between the melatonin receptor and ligand affinity and molecular structure, and the exploration of subtype selectivity (47–50).

Cluster 3 is related to the phase-shifting of melatonin. As the pace of life changes, the number of night shift workers increases, while many young people’s habits are reversed day and night. Because the body’s circadian rhythm signal is not synchronized with the external environment rhythm, work and rest time are disordered, insomnia and daytime sleepiness occur repeatedly, and the sleep-wake time shifts. When appropriate melatonin treatment is given at the proper time, melatonin will shift the sleep-wake time to where it should be. Melatonin can also reduce the impact of the outside world on the sleep-wake cycle. Because melatonin can adjust the phase shift and re-synchronize the endogenous rhythm and sleep-wake rhythm, it effectively promotes sleep. At the same time, melatonin can also be used to determine the stage of the body’s circadian rhythm, for example, through the urine melatonin metabolites 6-sulphatoxymelatonin (aMT6s). By monitoring the level of melatonin in night shift workers or others, it can be determined that their body is in different circadian rhythm periods (51–54).

Cluster 4 is related to seasonal reproduction. The reproductive cycle of most mammals is seasonal, and the hypothesis exists that the sexual cycle of mammals is related to the time of light and is regulated by the endogenous annual biological clock. Seasonal changes in an animal’s reproductive cycle are regulated by light-sensitive neural pathways. The changed photoperiod can regulate the hypothalamic-pituitary-gonadal axis (HPGA) by regulating the level of melatonin secretion. The periodic effect of light signal is closely related to deiodinase 2 and 3, RF amide-related peptide (RFRP), and kisspeptin. The suprachiasmatic nucleus (SCN), as the rhythm centre of mammals, is regulated by external light signals. The SCN indirectly regulates the thyroid hormone, RFRP system, and kisspeptin system through melatonin to regulate the function of the HPGA. These systems are for different sites of action in the hypothalamus, indicating that melatonin and the hypothalamus regulate the reproductive cycle in a variety of ways. These different hypothalamic sites are the key to reproductive regulation. The melatonin signal received by photoperiod regulation acts on the hypothalamus, thereby regulating the gonadotropin/gonad cycle, leading to the formation of the reproductive cycle. In addition, previous studies also found that melatonin can restore the neuroendocrine function of ageing ewes, which provides a new perspective and hope for the seasonal study of the reproductive cycle (55–58).

Cluster 5 is related to the regulation of melatonin secretion. As the body ages, although the pineal gland does not shrink significantly, its function declines sharply, resulting in a decrease in the level of melatonin secretion, which seriously affects the functions of many body systems. Restoring the physiological level of melatonin may delay the ageing process and will contribute to the treatment of melatonin-related diseases. Melatonin secretion is regulated by various aspects of the body. It was found that basal pineal melatonin levels in old rats are more sensitive to photoperiod changes than those in young rats, but isoproterenol can stimulate both young and old rat pineal glands irrespective of time or photoperiod, which shows that the melatonin response to isoproterenol is age-dependent and that the pineal gland response to isoproterenol is not photoperiod-dependent. There are many peptides related to melatonin secretion in the pineal gland. A peptide extract from the pineal epithelium can restore the level of melatonin secretion in the pineal gland, and a synthetic tetrapeptide has the same effect; however, the role of this peptide extract and isoproterenol in regulating melatonin secretion needs further study (59–61).

Cluster 6 is related to the anti-neurotoxin effect of melatonin. Studies have shown that melatonin can play a vital role in anti-neurotoxicity. Melatonin can resist the toxic effects of free radicals on brain tissue, such as hydroxyl free radicals, peroxy free radicals, singlet oxygen and toxic nitric oxide gas. In addition, melatonin can also activate a variety of antioxidant enzymes to play a role in neuroprotection and detoxification, such as superoxide dismutase, glutathione peroxidase and glutathione reductase. Melatonin has the advantage of crossing the blood-brain barrier and has no side effects. It is an excellent neuroprotective agent that has been considered a potential preventive and therapeutic drug for neurodegenerative diseases such as Parkinson’s disease (62–65).

Cluster 7 is related to the antioxidant properties of melatonin. Existing studies have found that melatonin has the effect of treating depression, tumors and osteoporosis, the mechanism of which is that melatonin has antioxidant functions and can protect biological macromolecules from oxidative damage. Melatonin protects cells through its direct free radical scavenging action, indirect antioxidant action, and cell membrane stabilization effect. Melatonin exerts its antioxidant effect mainly through its action on mitochondria. Studies have shown that melatonin can promote the mitochondrial electron transport chain, thereby preventing the generation of free radicals, reducing electron leakage, and preventing cell damage. Melatonin has the effect of resisting mitochondrial damage, preventing energy failure, and avoiding apoptosis. Moreover, melatonin has been shown to have a positive effect on physiological abnormalities caused by mitochondrial dysfunction in experiments and clinical diseases. In the future, we should deeply study the mechanism of melatonin action to treat diseases through different antioxidant mechanisms, clarify the target of melatonin action to treat different diseases, and develop new drugs to amplify the therapeutic effect of melatonin, which will be the focus of future research (66–69).

Our research also has certain limitations. Our study included only the literature published from 2000 to 2019, without literature published in 2020, and the citation analysis ended on June 16, without a progressive follow-up data update. However, over time, melatonin-related literature will be continuously updated in the database, which will have a certain impact on the real-time results of the bibliometric analysis and may cause some deviations between our results and the actual situation. Nevertheless, the deviations will not be very large, and the research results are still credible and reliable.

## Conclusions

Our results assessed the publication information regarding different countries, institutions, authors, journals, etc. and predict the future research hotspots and research trends on melatonin. The structure and normal physiological functions of melatonin have been intensively studied in the past few years. And clinical application research and target of melatonin treatment for different diseases and target-based drug design will certainly become the focus of melatonin research. Besides, reviewing previous high-cited melatonin studies help us to understand the authority in this field. Finally, clinical research on melatonin, especially randomized controlled trials, has great potential to guide the development of melatonin research in the future.

## Data Availability Statement

The original contributions presented in the study are included in the article/**Supplementary Material**. Further inquiries can be directed to the corresponding author.

## Author Contributions

YM and LT designed the overall study. YM and ZT carried out research, collected and analyzed data, and cowrote the draft. SZ and WD performed visualization. LT supervised this study. All authors contributed to the article and approved the submitted version.

## Funding

This work was supported by Natural Science Foundation of Liao Ning, grant number 2019-BS-294 and Construction of Clinical Medical Research Center of Orthopaedics and Sports Rehabilitation Diseases in Liaoning Province, grant number 2019416030.

## Conflict of Interest

The authors declare that the research was conducted in the absence of any commercial or financial relationships that could be construed as a potential conflict of interest.

## Publisher’s Note

All claims expressed in this article are solely those of the authors and do not necessarily represent those of their affiliated organizations, or those of the publisher, the editors and the reviewers. Any product that may be evaluated in this article, or claim that may be made by its manufacturer, is not guaranteed or endorsed by the publisher.
