# Supplementary material for: Research Hot Spots and Trends on Melatonin From 2000 to 2019
Source: Front Endocrinol (Lausanne). 2021 Nov 30;12:753923. doi: 10.3389/fendo.2021.753923 (PMC8669723; doi:10.3389/fendo.2021.753923)
Supplement: Supplementary file 1 [file Table_1.docx]

Table S1. Highly frequent Major MeSH terms/ MeSH subheadings terms from the included publications on melatonin.

| Rank | Major MeSH terms/ MeSH subheadings | Frequency | Proportion of frequency (%) | Cumulative percentage (%) |
| --- | --- | --- | --- | --- |
| 1 | Melatonin / pharmacology | 3703 | 6.6008 | 6.6008 |
| 2 | Melatonin / metabolism | 1640 | 2.9234 | 9.5242 |
| 3 | Melatonin / therapeutic use | 1469 | 2.6186 | 12.1428 |
| 4 | Circadian Rhythm / physiology | 1065 | 1.8984 | 14.0412 |
| 5 | Antioxidants / pharmacology | 939 | 1.6738 | 15.7151 |
| 6 | Melatonin / physiology | 687 | 1.2246 | 16.9397 |
| 7 | Melatonin / administration & dosage | 663 | 1.1818 | 18.1215 |
| 8 | Melatonin / blood | 607 | 1.082 | 19.2036 |
| 9 | Oxidative Stress / drug effects | 469 | 0.836 | 20.0396 |
| 10 | Melatonin / analogs & derivatives | 429 | 0.7647 | 20.8043 |
| 11 | Antioxidants / therapeutic use | 411 | 0.7326 | 21.5369 |
| 12 | Light | 400 | 0.713 | 22.25 |
| 13 | Circadian Rhythm | 392 | 0.6988 | 22.9487 |
| 14 | Photoperiod | 314 | 0.5597 | 23.5084 |
| 15 | Melatonin / biosynthesis | 314 | 0.5597 | 24.0682 |
| 16 | Pineal Gland / metabolism | 299 | 0.533 | 24.6012 |
| 17 | Sleep / physiology | 271 | 0.4831 | 25.0842 |
| 18 | Apoptosis / drug effects | 266 | 0.4742 | 25.5584 |
| 19 | Circadian Rhythm / drug effects | 246 | 0.4385 | 25.9969 |
| 20 | Pineal Gland / physiology | 193 | 0.344 | 26.3409 |
| 21 | Neuroprotective Agents / pharmacology | 187 | 0.3333 | 26.6743 |
| 22 | Melatonin / chemistry | 171 | 0.3048 | 26.9791 |
| 23 | Antioxidants / metabolism | 165 | 0.2941 | 27.2732 |
| 24 | Melatonin / analysis | 164 | 0.2923 | 27.5656 |
| 25 | Seasons | 163 | 0.2906 | 27.8561 |
| 26 | Sleep Initiation and Maintenance Disorders / drug therapy | 150 | 0.2674 | 28.1235 |
| 27 | Sleep / drug effects | 149 | 0.2656 | 28.3891 |
| 28 | Aging / physiology | 148 | 0.2638 | 28.6529 |
| 29 | Sleep Wake Disorders / drug therapy | 144 | 0.2567 | 28.9096 |
| 30 | Brain / drug effects | 124 | 0.221 | 29.1306 |
| 31 | Free Radical Scavengers / pharmacology | 124 | 0.221 | 29.3517 |
| 32 | Signal Transduction / drug effects | 123 | 0.2193 | 29.5709 |
| 33 | Circadian Rhythm / radiation effects | 120 | 0.2139 | 29.7848 |
| 34 | Liver / drug effects | 116 | 0.2068 | 29.9916 |
| 35 | Antioxidants / administration & dosage | 115 | 0.205 | 30.1966 |
| 36 | Receptor, Melatonin, MT1 / metabolism | 115 | 0.205 | 30.4016 |
| 37 | Melatonin / urine | 113 | 0.2014 | 30.603 |
| 38 | Lipid Peroxidation / drug effects | 113 | 0.2014 | 30.8045 |
| 39 | Brain / metabolism | 112 | 0.1996 | 31.0041 |
| 40 | Central Nervous System Depressants / therapeutic use | 100 | 0.1783 | 31.1824 |
| 41 | Neurons / drug effects | 97 | 0.1729 | 31.3553 |
| 42 | Receptors, Melatonin / metabolism | 97 | 0.1729 | 31.5282 |
| 43 | Aging / metabolism | 95 | 0.1693 | 31.6975 |
| 44 | Mitochondria / metabolism | 91 | 0.1622 | 31.8597 |
| 45 | Biological Clocks / physiology | 89 | 0.1586 | 32.0184 |
| 46 | Receptor, Melatonin, MT2 / metabolism | 85 | 0.1515 | 32.1699 |
| 47 | Hippocampus / drug effects | 84 | 0.1497 | 32.3196 |
| 48 | Reactive Oxygen Species / metabolism | 83 | 0.148 | 32.4676 |
| 49 | Hypnotics and Sedatives / therapeutic use | 81 | 0.1444 | 32.612 |
| 50 | Oxidative Stress | 81 | 0.1444 | 32.7564 |
| 51 | Neuroprotective Agents / therapeutic use | 80 | 0.1426 | 32.899 |
| 52 | Mitochondria / drug effects | 79 | 0.1408 | 33.0398 |
| 53 | Circadian Rhythm / genetics | 76 | 0.1355 | 33.1753 |
| 54 | Sleep Disorders, Circadian Rhythm / drug therapy | 76 | 0.1355 | 33.3108 |
| 55 | Pineal Gland / drug effects | 74 | 0.1319 | 33.4427 |
| 56 | Reperfusion Injury / prevention & control | 71 | 0.1266 | 33.5692 |
| 57 | Melatonin / pharmacokinetics | 69 | 0.123 | 33.6922 |
| 58 | Reproduction / physiology | 68 | 0.1212 | 33.8134 |
| 59 | Sheep / physiology | 68 | 0.1212 | 33.9347 |
| 60 | Serotonin / metabolism | 68 | 0.1212 | 34.0559 |
| 61 | Antineoplastic Agents / pharmacology | 66 | 0.1176 | 34.1735 |
| 62 | Melatonin | 66 | 0.1176 | 34.2912 |
| 63 | Work Schedule Tolerance / physiology | 66 | 0.1176 | 34.4088 |
