# Supplementary material for: Research Hot Spots and Trends on Melatonin From 2000 to 2019
Source: Front Endocrinol (Lausanne). 2021 Nov 30;12:753923. doi: 10.3389/fendo.2021.753923 (PMC8669723; doi:10.3389/fendo.2021.753923)
Supplement: Supplementary file 2 [file Table_2.docx]

Table S2. The 100 most-cited articles on melatonin research.

| No. | Title | Total  Citation |
| --- | --- | --- |
| 1 | The Movement Disorder Society Evidence-Based Medicine Review Update: Treatments for the Non-Motor Symptoms of Parkinson's Disease | 448 |
| 2 | Gastrointestinal melatonin: Localization, function, and clinical relevance | 426 |
| 3 | Effects of melatonin treatment in septic newborns | 350 |
| 4 | Practice parameters for the clinical evaluation and treatment of circadian rhythm sleep disorders | 310 |
| 5 | International Union of Basic and Clinical Pharmacology. LXXV. Nomenclature, Classification, and Pharmacology of G Protein-Coupled Melatonin Receptors | 284 |
| 6 | Circadian rhythm sleep disorders: Part 1, basic principles, shift work and jet lag disorders | 278 |
| 7 | CASE HISTORY Agomelatine, the first melatonergic antidepressant: discovery, characterization and development | 266 |
| 8 | Human pineal physiology and functional significance of melatonin | 258 |
| 9 | Should supplemental antioxidant administration be avoided during chemotherapy and radiation therapy? | 257 |
| 10 | Melatonin: From basic research to cancer treatment clinics | 247 |
| 11 | Circadian rhythm disturbances in depression | 237 |
| 12 | Hypothalamic stimulation in chronic cluster headache: a pilot study of efficacy and mode of action | 246 |
| 13 | Circadian rhythm sleep disorders: Part II, advanced sleep phase disorder, delayed sleep phase disorder, free-running disorder, and irregular sleep-wake rhythm | 232 |
| 14 | British Association for Psychopharmacology consensus statement on evidence-based treatment of insomnia, parasomnias and circadian rhythm disorders | 228 |
| 15 | Melatonin as a radioprotective agent: A review | 215 |
| 16 | STRESS AND THE GUT: PATHOPHYSIOLOGY, CLINICAL CONSEQUENCES, DIAGNOSTIC APPROACH AND TREATMENT OPTIONS | 202 |
| 17 | A multicenter, placebo-controlled trial of melatonin for sleep disturbance in Alzheimer's disease | 213 |
| 18 | Wrist Actigraphy | 188 |
| 19 | Melatonin: An Established Antioxidant Worthy of Use in Clinical Trials | 205 |
| 20 | Oxidative stress of the newborn in the pre- and postnatal period and the clinical utility of melatonin | 203 |
| 21 | Efficacy of agomelatine, a MT1/MT2 receptor agonist with 5-HT2C antagonistic properties, in major depressive disorder | 199 |
| 22 | Clinical Uses of Melatonin: Evaluation of Human Trials | 188 |
| 23 | Role of the Toll Like Receptor (TLR) Radical Cycle in Chronic Inflammation: Possible Treatments Targeting the TLR4 Pathway | 185 |
| 24 | Melatonin protects against common deletion of mitochondrial DNA-augmented mitochondrial oxidative stress and apoptosis | 194 |
| 25 | Serotonin 5-HT2C receptors as a target for the treatment of depressive and anxious states: Focus on novel therapeutic strategies | 191 |
| 26 | Excessive sleepiness in adolescents and young adults: Causes, consequences, and treatment strategies | 189 |
| 27 | European guideline for the diagnosis and treatment of insomnia | 128 |
| 28 | Clinical Practice Guideline: Tinnitus | 173 |
| 29 | Randomized, double-blind clinical trial, controlled with placebo, of the toxicology of chronic melatonin treatment | 179 |
| 30 | Broad targeting of angiogenesis for cancer prevention and therapy | 169 |
| 31 | Interventions for preventing deliriumin hospitalised non-ICU patients | 156 |
| 32 | Parkinson risk in idiopathic REM sleep behavior disorder Preparing for neuroprotective trials | 169 |
| 33 | Role of hippocampal oxidative stress in memory deficits induced by sleep deprivation in mice | 176 |
| 34 | Clinical Practice Guideline for the Pharmacologic Treatment of Chronic Insomnia in Adults: An American Academy of Sleep Medicine Clinical Practice Guideline | 146 |
| 35 | Improvement in subjective sleep in major depressive disorder with a novel antidepressant, agomelatine: Randomized, double-blind comparison with venlafaxine | 178 |
| 36 | Efficacy of the Novel Antidepressant Agomelatine on the Circadian Rest-Activity Cycle and Depressive and Anxiety Symptoms in Patients With Major Depressive Disorder: A Randomized, Double-Blind Comparison With Sertraline | 169 |
| 37 | The potential of melatonin in reducing morbidity-mortality after craniocerebral trauma | 167 |
| 38 | Melatonin: Both master clock output and internal time-giver in the circadian clocks network | 160 |
| 39 | Oxidative mechanisms and tardive dyskinesia | 163 |
| 40 | Strategies for safe and effective therapeutic measures for chronic arsenic and lead poisoning | 157 |
| 41 | Melatonin: Characteristics, concerns, and prospects | 153 |
| 42 | Modified-Release Hydrocortisone to Provide Circadian Cortisol Profiles | 151 |
| 43 | Practice Parameter: Treatment of nonmotor symptoms of Parkinson disease Report of the Quality Standards Subcommittee of the American Academy of Neurology | 151 |
| 44 | Melatonin as a potential antihypertensive treatment | 149 |
| 45 | Reduced oxidative damage in ALS by high-dose enteral melatonin treatment | 144 |
| 46 | Andropause: A misnomer for a true clinical entity | 149 |
| 47 | Melatonin and its analogs in insomnia and depression | 139 |
| 48 | Melatonin Antioxidative Defense: Therapeutical Implications for Aging and Neurodegenerative Processes | 140 |
| 49 | Clinical, anatomical, and physiologic relationship between sleep and headache | 141 |
| 50 | Melatonin augments hypothermic neuroprotection in a perinatal asphyxia model | 135 |
| 51 | Past and future approaches to ischemia-reperfusion lesion associated with liver transplantation | 137 |
| 52 | Agomelatine Prevents Relapse in Patients With Major Depressive Disorder Without Evidence of a Discontinuation Syndrome: A 24-Week Randomized, Double-Blind, Placebo-Controlled Trial | 134 |
| 53 | Antioxidant strategies for Alzheimer's disease | 131 |
| 54 | The neurobiology of depression | 119 |
| 55 | Characteristics of menstrual and nonmenstrual attacks in women with menstrually related migraine referred to headache centres | 128 |
| 56 | Melatonin, sleep, and circadian rhythms: rationale for development of specific melatonin agonists | 130 |
| 57 | Impact of antioxidant supplementation on chemotherapeutic toxicity: A systematic review of the evidence from randomized controlled trials | 129 |
| 58 | Melatonin-dopamine interactions: From basic neurochemistry to a clinical setting | 125 |
| 59 | Bright Light Treatment in Elderly Patients With Nonseasonal Major Depressive Disorder A Randomized Placebo-Controlled Trial | 122 |
| 60 | When melatonin gets on your nerves: Its beneficial actions in experimental models of stroke | 125 |
| 61 | The utility of melatonin in reducing cerebral damage resulting from ischemia and reperfusion | 121 |
| 62 | Prolonged-release melatonin improves sleep quality and morning alertness in insomnia patients aged 55 years and older and has no withdrawal effects | 120 |
| 63 | The inflammatory process of gout and its treatment | 121 |
| 64 | Clinical trials of controlled-release melatonin in children with sleep-wake cycle disorders | 122 |
| 65 | Impact of antioxidant supplementation on chemotherapeutic efficacy: A systematic review of the evidence from randomized controlled trials | 120 |
| 66 | Relative abuse liability of hypnotic drugs: A conceptual framework and algorithm for differentiating among compounds | 122 |
| 67 | Biomarkers in Parkinson's disease (recent update) | 112 |
| 68 | Melatonin reduces oxidative stress in surgical neonates | 116 |
| 69 | Medication use in the treatment of pediatric insomnia: Results of a survey of community-based pediatricians | 119 |
| 70 | Anti-angiogenic activity of melatonin in advanced cancer patients | 118 |
| 71 | Efficacy of prolonged release melatonin in insomnia patients aged 55-80 years: quality of age sleep and next-day alertness outcomes | 114 |
| 72 | Hypnic headache - Clinical features, pathophysiology, and treatment | 118 |
| 73 | Melatonin-enhanced autophagy protects against neural apoptosis via a mitochondrial pathway in early brain injury following a subarachnoid hemorrhage | 112 |
| 74 | Bright-Light Therapy in the Treatment of Mood Disorders | 108 |
| 75 | Therapeutic treatments potentially mediated by melatonin receptors: potential clinical uses in the prevention of osteoporosis, cancer and as an adjuvant therapy | 114 |
| 76 | The Use of Exogenous Melatonin in Delayed Sleep Phase Disorder: A Meta-analysis | 108 |
| 77 | A two-part, double-blind, placebo-controlled trial of exogenous melatonin in REM sleep behaviour disorder | 107 |
| 78 | Melatonin, a Full Service Anti-Cancer Agent: Inhibition of Initiation, Progression and Metastasis | 97 |
| 79 | A practical approach to circadian rhythm sleep disorders | 109 |
| 80 | Decreased MT1 melatonin receptor expression in the suprachiasmatic nucleus in aging and Alzheimer's disease | 108 |
| 81 | Five years survival in metastatic non-small cell lung cancer patients treated with chemotherapy alone or chemotherapy and melatonin: a randomized trial | 109 |
| 82 | Synthesis of a novel series of tricyclic indan derivatives as melatonin receptor agonists | 109 |
| 83 | A review of melatonin as a suitable antioxidant against myocardial ischemia-reperfusion injury and clinical heart diseases | 104 |
| 84 | Skin deep: enhanced sleep depth by cutaneous temperature manipulation | 109 |
| 85 | Efficacy of agomelatine in generalized anxiety disorder: A randomized, double-blind, placebo-controlled study | 106 |
| 86 | Melatonin for insomnia in children with autism spectrum disorders | 106 |
| 87 | Acupuncture increases nocturnal melatonin secretion and reduces insomnia and anxiety: A preliminary report | 103 |
| 88 | Circadian Rhythm Profiles in Women with Night Eating Syndrome | 103 |
| 89 | American Geriatrics Society Abstracted Clinical Practice Guideline for Postoperative Delirium in Older Adults | 95 |
| 90 | Melatonin treatment for tardive dyskinesia - A double-blind, placebo-controlled, crossover study | 102 |
| 91 | The role of melatonin as an antioxidant in the follicle | 92 |
| 92 | Melatonin Improves Glucose Homeostasis and Endothelial Vascular Function in High-Fat Diet-Fed Insulin-Resistant Mice | 100 |
| 93 | Long-term follow-up of melatonin treatment in children with ADHD and chronic sleep onset insomnia | 98 |
| 94 | Sleep in the critically ill patient | 97 |
| 95 | Oxidative and inflammatory parameters in respiratory distress syndrome of preterm newborns: Beneficial effects of melatonin | 99 |
| 96 | Clinical Aspects of Melatonin Intervention in Alzheimer's Disease Progression | 97 |
| 97 | Randomized clinical trial of bright light therapy for antepartum depression: Preliminary findings | 100 |
| 98 | Melatonin in Alzheimer's Disease | 90 |
| 99 | Rapid eye movement sleep behavior disorder: devising controlled active treatment studies for symptomatic and neuroprotective therapy-a consensus statement from the International Rapid Eye Movement Sleep Behavior Disorder Study Group | 95 |
| 100 | Melatonin protects against oxidative organ injury in a rat model of sepsis | 91 |
