# Supplementary material for: Research Hot Spots and Trends on Melatonin From 2000 to 2019
Source: Front Endocrinol (Lausanne). 2021 Nov 30;12:753923. doi: 10.3389/fendo.2021.753923 (PMC8669723; doi:10.3389/fendo.2021.753923)
Supplement: Supplementary file 3 [file Table_3.docx]

Table S3. Completed phase II clinical trials of melatonin

|  | Study Title | Conditions | Interventions |
| --- | --- | --- | --- |
| 1 | Melatonin in Smoke-induced Vascular Injury | Smoking Vascular System Injuries | Other: non-smoker oral placebo Drug: non-smoker oral melatonin Other: smoker oral placebo Drug: smoker oral melatonin |
| 2 | Melatonin Effect in Combination With Neoadjuvant Chemotherapy to Clinical Response in Locally Advanced Oral Squamous Cell Carcinoma | Oral Squamous Cell Carcinoma Neoadjuvant Chemotherapy | Drug: Melatonin 20 MG Oral Capsule Drug: Placebo oral capsule |
| 3 | Melatonin Intervention For Neurocognitive Deficits in the St. Jude Lifetime Cohort | Cancer Malignancies | Drug: melatonin Drug: placebo |
| 4 | Melatonin As A Novel Neuroprotectant In Preterm Infants- Dosage Study | Premature Birth Brain Injury | Drug: Melatonin injection |
| 5 | Functional Melatonin Replacement for Sleep Disruptions in Individuals With Tetraplegia | Insomnia Spinal Cord Injury Tetraplegia Sleep Disorders | Drug: Ramelteon Drug: Placebo |
| 6 | Melatonin Osteoporosis Prevention Study | Osteoporosis Osteopenia | Dietary Supplement: melatonin Dietary Supplement: sugar pill |
| 7 | Melatonin for Fatigue and Other Symptoms in Patients With Advanced Cancer | Cancer Fatigue | Drug: Melatonin Drug: Placebo |
| 8 | Melatonin and Nighttime Blood Pressure in African Americans-8 mg Study | Hypertension | Drug: Melatonin Drug: Placebo |
| 9 | Melatonin and Nighttime Blood Pressure in African Americans--24 mg Study | Hypertension | Drug: Melatonin Drug: Placebo |
| 10 | Premedication With Melatonin and Alprazolam Combination Versus Alprazolam or Melatonin Alone | Anxiety | Drug: meloset (melatonin) Drug: stresnil (melatonin and alprazolam) Drug: (alprax) alprazolam Drug: placebo |
| 11 | A Study of the Efficacy of Prolonged-Release Melatonin Versus Placebo in Diabetic Patients Suffering From Insomnia | Diabetes Mellitus, Type 2 Insomnia | Drug: Circadin Drug: Placebo |
| 12 | Effects of Melatonin on Insomnia Symptoms in Older Adults | Sleep Initiation and Maintenance Disorders | Drug: Melatonin 0.4 mg Drug: Melatonin 4.0 mg Drug: Placebo |
| 13 | Effects of Kuvan on Melatonin Secretion | Phenylketonuria (PKU) | Drug: Kuvan Dietary Supplement: Large Neutral Amino Acid Therapy |
| 14 | Melatonin and the Metabolic Syndrome | Metabolic Syndrome | Drug: Melatonin Drug: Placebo |
| 15 | Melatonin Versus Placebo and the Effect on Appetite in Advanced Cancer Patients | Gastrointestinal Cancer Lung Cancer | Drug: Melatonin Drug: Placebo |
| 16 | Melatonin Versus Placebo in Breast Cancer | Breast Cancer | Drug: Melatonin 3 mg |
| 17 | Melatonin Supplements for Improving Sleep in Individuals With Hypertension | Sleep Disorders Hypertension | Drug: Melatonin Drug: Placebo |
| 18 | Treatment of Osteopenia With Melatonin | Osteoporosis | Drug: Melatonin |
| 19 | Study of Melatonin on Sleep, Pain, and Confusion After Joint Replacement Surgery | Postoperative Pain Delirium Confusion Sleep | Drug: Melatonin Drug: Placebo |
| 20 | Mechanisms of Sleep Latency and Health: The Effect of a Melatonin Receptor Agonist in Inflammation and Insulin Resistance | Insomnia | Drug: ramelteon Drug: placebo |
| 21 | Efficacy and Safety of Circadin® 2 mg in the Treatment of Primary Insomnia Patients | Primary Insomnia | Drug: Circadin Drug: placebo circadin |
| 22 | Efficacy of Circadin® 2 mg in Patients With Mild to Moderate Alzheimer Disease Treated With AChE Inhibitor | Alzheimer's Disease Sleep Disorder | Drug: Circadin Drug: Placebo |
| 23 | Efficacy and Safety of Circadin® in the Treatment of Sleep Disturbances in Children With Neurodevelopment Disabilities | Sleep Disorders | Drug: Circadin 2/5/10 mg Drug: Placebo |
| 24 | Melatonin Agonist Effects of Tasimelteon Versus Placebo in Patients With Major Depressive Disorder | Major Depressive Disorder | Drug: tasimelteon Drug: placebo |
| 25 | Randomized Controlled Trial on the Treatment Effects of Melatonin and Light Therapy on Delayed Sleep Phase Syndrome | Delayed Sleep Phase Syndrome | Dietary Supplement: Melatonin Other: Bright light Other: Placebo red light Other: Placebo capsule |
| 26 | [Efficacy and Safety Study of a Combination Product [Drug:BCI-024 (Buspirone) and Drug:BCI-049 (Melatonin)] to Treat Major Depressive Disorder (MDD)](https://clinicaltrials.gov/ct2/show/NCT00705003?recrs=e&rslt=With&cond=Melatonin&draw=4&rank=26) | Major Depressive Disorder | Drug: BCI-024: over-encapsulated Buspirone tablet 15 mg QD and BCI-049: over-encapsulated Melatonin tablet 3 mg QD Drug: BCI-024 (Buspirone) Drug: Matching placebo |
| 27 | Effect of Ramelteon on Smoking Abstinence | Tobacco Use Disorder | Drug: Ramelteon Drug: Placebo |
| 28 | Individually Tailored Lighting System to Improve Sleep in Older Adults | Sleep Disturbances | Other: Blue light Other: Red light |
| 29 | Pharmacotherapy and Mechanisms of Sleep Disturbance in Alcohol Dependence | Alcohol Dependence Insomnia | Drug: Placebo dispensed to subject. Drug: Gabapentin dispensed to subject. |
| 30 | Multiple Dose Study Of PF-05251749 In Healthy Volunteers | Healthy Adult Subjects Healthy Elderly Subjects | Drug: PF-05251749 Drug: Melatonin |
